# Barriers and Facilitators of Older Workers' Abilities to Obtain and Maintain Employment: A Scoping Review

**DOI:** 10.1155/jare/5609589

**Published:** 2025-11-25

**Authors:** Jonathan Lai, Jacquie Eales, Matthew Lariviere, Jennifer Boger, Janet Fast

**Affiliations:** ^1^Department of Human Ecology, Faculty of Agricultural, Life, and Environmental Sciences, University of Alberta, 116 St & 85 Ave, Edmonton T6G-2R3, Alberta, Canada; ^2^School of Healthcare and Nursing Sciences, Faculty of Health and Wellbeing, Northumbria University, 15 Coach Lane, Tyne and Wear, Newcastle-upon-Tyne NE7 7TR, UK; ^3^Department of Systems Design Engineering, Faculty of Engineering, University of Waterloo, 200 University Avenue West, Waterloo N2L-3G1, Ontario, Canada

**Keywords:** employability, employment barriers and facilitators, extending working lives, older workers, scoping review

## Abstract

Throughout adulthood, the ability to access employment is vital for financial well-being, social inclusion and civic participation. This scoping review explores the factors that facilitate or challenge the abilities of workers age 50 and older to obtain and maintain paid employment. A total of 244 academic and grey literature articles were included in this scoping review. To frame the data extraction and analysis of included literature, we drew on Human Ecology Theory, a multidisciplinary theory that posits that individuals affect and are affected by the contexts they inhabit. Four key contexts were identified that impact older workers' employability (and the relevant codes that comprise each context): individual context (health, income and wealth accumulation, education and skills, employment history, lifestyle preferences and personal characteristics); family context (obligations with intimate partners, obligations to dependent children and caregiving obligations); workplace context (organizational characteristics, workplace policies, job characteristics and workplace relationships) and sociopolitical context (ageism, government labour and pension policies and macroeconomic conditions). We conclude that the employability of older workers is not attributable to a single factor within any of these contexts. Rather, older workers' abilities to maintain their labour force participation are fluid, situational and temporal, including aspects that arise across a person's life course as forms of cumulative advantage or disadvantage. Policies to support older workers' labour force participation require governments and employers to recognize how the dynamic lived experiences and relationships of individuals—within families, workplaces and society—shape their employability in later life.

## 1. Introduction

Regardless of one's age, the ability to access employment is not only important for financial reasons but also for personal identity and self-esteem and is vital to social inclusion and civic participation [1]. The United Nations (UN) also recognizes the importance of decent work as part of inclusive and sustained economic growth in eradicating poverty and enhancing well-being. The 193 UN member states from across the globe are committed to ensuring all persons have access to employment and good quality jobs as part of its 2030 Agenda for Sustainable Development [2], and promise of leaving no one behind [3].

While the term ‘longevity revolution' coined by Butler [4] is worth celebrating as a society, longer lifespans equate to a greater need for individuals to have adequate financial security to support their later-life financial well-being than in previous generations. Across countries that comprise the Organisation for Economic Co-operation and Development (OECD), average life expectancies have risen since the 1960s to 81 years [5]. One of the fastest growing age groups are those 85 and older [6], and the number of centenarians, globally, has now surpassed half a million [7].

Yet, in recent decades, population ageing has been used often as a rationale by governments of high-income countries to dismantle public programs [8]. Policies that once promoted early retirement around age 60 in the 1990s as a lever to address unemployment have been reversed in many countries. Extended working life policies, which delay the age an individual becomes eligible for public pensions, have been encouraged across governments in Europe, Australia, New Zealand, and the United States to sustain retirement systems [9] and maintain strong labour markets [10, 11]. Critics of extended working life policies contend that this ‘one-size fits all' approach ignores the diversity of older people living and earning longer [9], and risks plunging older people into poverty [12]. In the context of retrenched welfare states, increased life expectancies pose financial risks for individuals who lack sufficient personal savings and wealth that enable choice in whether or not to work in later life [4].

While considerable research has been conducted about the employment of older workers across many disciplines, only one study has sought to map the literature on this topic. A previous systematic mapping review by Edge et al. [13] explored the barriers and facilitators to extending working lives of older women in Europe. Their review, which included 15 English-language studies published between 2005 and 2016, was restricted to countries within the European Union and European Economic Areas. They found that decisions to retire or extend working lives were linked to health, social factors, workplace factors, financial security and pension systems.

As older workers continue to comprise an increasing proportion of the global labour force [14], there is a growing need to understand the elements that enable or impede their employability. Employability is a multidimensional construct and is influenced by many individual, interpersonal, organizational, policy, cultural and societal factors [15]. The purpose of this scoping review was to explore if the factors identified by Edge et al. [13] would extend beyond the boundaries of Europe and examine the extent of literature associated with older worker employability. The research question underpinning our study was as follows: What are the barriers and facilitators of older workers' abilities to obtain and maintain paid employment?

## 2. Methods

We used a scoping review methodology [16, 17] to explore and examine available evidence in a systematic manner [18]. The strength of scoping reviews is in their comprehensive treatment of a large, complex or heterogeneous body of existing literature [19]. We followed the Joanna Briggs Institute methodological framework [20] and sought guidance on our search strategy from a subject expert librarian at a public university in Canada.

### 2.1. Inclusion Criteria

Our inclusion criteria defined the boundaries of the study and informed an effective search strategy. We defined the population of interest as older workers and older job seekers. While there is not a consistent agreed upon age at which a person becomes an ‘older worker' [21], we defined older workers as being aged 50 and older. Studies with participants under age 50 were included if the mean age of the sample was at least 50 years old, or if studies made comparisons between workers under age 50 and older workers. We included studies that examined factors that created opportunities or barriers to paid employment for older workers and older job seekers, including labour force exits that may be described as involuntary and forced by circumstances beyond an older worker's control [22].

Scoping reviews draw on evidence from any research methodology (quantitative, qualitative, mixed methods or literature reviews) and may also include evidence from nonresearch sources [20]. For this review, the literature included peer-reviewed articles published in academic journals or grey literature reports produced by bodies such as governments, businesses and nongovernmental organizations [23]. The publication date range of 1 January 2005 and 30 June 2020 was chosen in consultation with our librarian. This 15-year time frame would capture contemporary knowledge on this topic while excluding the unprecedented impact the COVID-19 pandemic had on older workers' labour force participation, particularly among women [24]. Articles were excluded if they were published before 2005, whole books, not published in English, study protocols, journal issue introduction papers, essays or white-papers, macroeconometric articles, did not have an appropriate sample of older workers or job seekers, had no relevant employability findings, or only had descriptive findings with no inferential analysis conducted.

### 2.2. Search Strategies

Seven academic databases were searched: Academic Search Complete, Business Source Complete, CINAHL, MEDLINE, PsycINFO, Scopus and SocINDEX with Full Text. The key search terms and search string strategy are detailed in Table 1. Recognizing that the employability of older workers is of interest to governments, advocacy groups and not-for-profit organizations, we also reviewed the organizational websites of AARP (USA), CARP (Canada), Eurofound (Europe) and two global organizations: OECD and the World Health Organization (WHO) to identify relevant reports about older workers. In addition, we searched Canadian Research Index and DesLibris databases for Canadian policy documents and reports on older workers using the search term older worker^∗^. To include coverage of grey literature from Oceania, we conducted a Google Advance Search using the search terms older worker^∗^ AND Australia and older worker^∗^ AND ‘New Zealand'. A decision was made a priori to screen only the first 30 hits, determined by search engine relevance, to keep the scope of these searches manageable.

**Figure 1:**
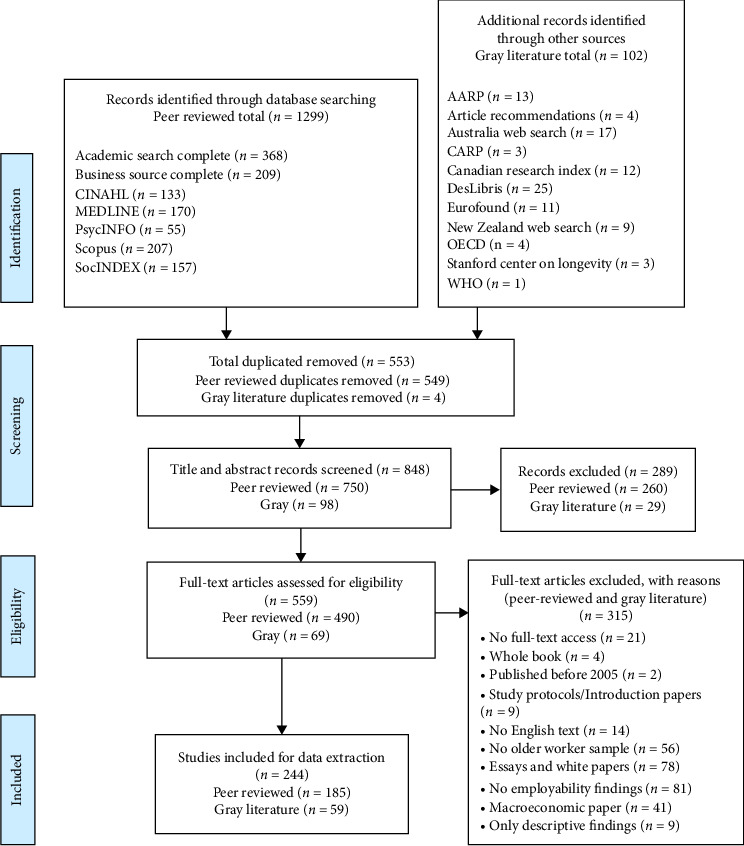
PRISMA-SCR flow diagram.

To prepare for the literature screening, we used a two-stage process to ensure inter-rater reliability at the title and abstract screening stage and the full-text review stage to verify that the inclusion and exclusion criteria were applied consistently by two reviewers [20]. At the first stage, a sample of 50 titles and abstracts were reviewed independently by two of the authors; decisions to include studies for full-text review were compared and any discrepancies were discussed. The title and abstract screening stage began after the two authors reached an inter-rater agreement of 80% or greater. At the second stage, the same two authors read independently and compared 5% of full-text articles to once again test consistency in applying the inclusion and exclusion criteria. Any uncertainties about study inclusion were resolved through discussion. The full-text review stage began after the two authors had an inter-rater reliability of 80% or greater. One author was responsible for the entire full-text screening. Details of the screening process are documented in the PRISMA-SCR diagram shown in Figure 1.

### 2.3. Data Extraction

One of the key challenges of conducting a scoping review lies in charting and summarizing complex concepts in a meaningful way [17]. With 244 articles to read and extract data from, we anticipated identifying a multitude of factors that enabled or impeded older workers' ability to secure and maintain paid employment. To manage this process, we needed a working analytic framework and used Coggle (https://coggle.it) to map concepts as we read the included articles and labelled factors that enabled or impeded older workers' employability (referred to as codes). To organize these codes into an analytic framework, we drew on the assumptions of Human Ecology Theory, a multidisciplinary theory that posits that individuals affect and are affected by the contexts they inhabit [25]. Applied to older workers, we sorted the codes into four broad contexts: the individual or personal context of older workers; the older workers' family context; the older workers' workplace context and the broader sociopolitical context in which older workers live. The concept mapping underwent several iterations, reflecting a nonlinear interplay between data extraction, coding and analysis. These contexts, and the codes that comprise them, are not discrete categories, but rather, demonstrate the dynamic environments, relationships and life course events that affect older workers' employability. Using the concept map as an initial analytic framework, we then entered data into an MS Excel spreadsheet to facilitate the data extraction and narrative processes [26]. Extracted data were citation information (authors, publication source, year of publication), information about the study sample and methods (country and region of origin, size of sample and type of study) and relevant findings sorted into the four established contexts and their associated codes.

### 2.4. Findings

The 244 included articles spanned a wide variety of disciplines, methods and geographic regions. Disciplines included social gerontology, social sciences, health sciences, business and human resource management. Methods from these studies included: quantitative (165), qualitative (28), mixed methods (11), review or synthesis of literature (9), policy analysis (5) and others (13). The represented regions were: Europe (140), North America (66), Oceania (24), Asia (6) and Latin America (1); seven articles were multiregional; Africa was not mentioned in any of the included literature. Over half of the included articles (55%) were published in 2014 or later.

Table 2 summarizes the four key contexts that influence employability of older workers, and relevant codes that comprise each context: individual (health, income and wealth accumulation, education and skills, employment history, lifestyle preferences and personal characteristics); family (obligations with intimate partners, obligations to dependent children and caregiving obligations and responsibilities); workplace (organizational characteristics, workplace policies and programs, job characteristics and workplace relationships) and sociopolitical (ageism, government labour and pension policies and macroeconomic conditions) contexts. Below, we elaborate on how each context influences the ability of older workers to find and maintain paid employment, noting the relative breadth of knowledge in each context.

#### 2.4.1. Context 1: Individual Context of Older Workers

##### 2.4.1.1. Older Workers' Health

Poor health had strong associations with personal decisions to leave the labour force and exits that were more forced and involuntary in nature. Poorly perceived health status, chronic illnesses (including depression, heart disease, stroke, diabetes, arthritis, poor sleep quality), multimorbidity, activity limitations and poor quality of life were associated with reduced labour force participation [27, 28], unemployment [27, 29–31], early retirement [29, 32, 33], intentions to retire early [34, 35], retiring because of a disability [28, 36] and becoming self-employed [37]. Having symptoms of depression, anxiety or other mental health issues were significant predictors of a work-related disability [38, 39] and job exit [40, 41].

Health issues were relevant in industries where individuals relied on their physical abilities for work, such as construction and engineering [42]. Health was also a consideration when deciding to return to the labour market following illness [43, 44]. For example, people were more likely to return to paid work following absence with a less advanced stage of cancer [44]. High rates of sickness absenteeism predicted job termination among older (but not younger) employees [45].

The desire to keep physically and mentally active was a common reason for individuals to continue working [46, 47], and active lifestyles supported older workers' physical capacity for work [48]. Lack of physical activity and obesity were strongly associated with withdrawal from the labour force [29, 49]. Risk behaviours, such as high alcohol consumption or drug addiction, were also associated with unemployment [50], early work exit [51–54] and retirement [49, 55].

##### 2.4.1.2. Older Workers' Income and Wealth Accumulation

Financial security and having sufficient accumulated wealth were significantly correlated with older workers' employment and retirement decisions [56, 57]. Having higher income, pension or accumulated wealth increased the likelihood of intentions, decisions and actions to retire early [35, 52, 56, 58–63], or become self-employed [64] and lowered the likelihood of retirement reversals [65, 66]. Decisions to retire early were also linked to financial incentives offered by employers [59, 62].

In contrast, older workers with income constraints, personal debt or insufficient retirement pension income were inclined to work longer [64, 67, 68], often beyond the typical retirement age of 65 [13, 59, 69, 70], or return to work following a first retirement [71]. Financial loss was a significant predictor of interest in returning to work among retirees under age 65 [72].

Displaced older workers who secured new jobs often earned less (between 8% and 17% on average) than their previous job [73]. Low compensation or unstable employment forced some older workers to leave their jobs in search of better paid or more stable employment [40, 74, 75]. Older women's employment decisions were influenced by household or spousal income [76–78]. Older workers with access to stable income from a partner or other sources were more likely to exit the labour force [31, 77].

##### 2.4.1.3. Older Workers' Education and Skills

Level of formal education attained was a predictor of older workers' employability and early retirement intentions [79, 80]. A study of 10 European countries found that lower educational attainment (high school diploma or less) was significantly associated with early labour force exit among those aged 50 to 65 [29, 81]. Older workers with low educational levels were at greater risk of exiting from work due to disability [53], having little choice or control over the decision to retire [82, 83] and experiencing long-term unemployment [84]. Women who had less than high school education had a higher risk of exiting the labour force because of a work disability [85].

Compared to those with high school education or less, persons with post-secondary education had a greater likelihood of remaining in the labour market [32, 78, 86–91], working longer [13, 47, 92], returning to work following an illness [44, 61], regaining employment following a job loss [93] and becoming self-employed [37, 64]. Moreover, those with a post-secondary degree were also more likely to have greater control in when they retired, often retiring earlier than those without a degree [64, 80] although the association was not strong [94]; this may be because they can afford to do so.

While education level seems to affect labour force participation among older workers, it does not appear to correlate well with job re-entry. Some older trade workers who have specific, transferable in-demand skills often found it easier to find new jobs than some highly educated workers [95]. Yet, overly specialized expertise was found to be a potential barrier to labour market reintegration [96], forcing some mature job seekers to omit post-graduate degrees and specialized training from their resumes [95]. Some older job seekers were disadvantaged by their relative lack of technological expertise [43], digital skills [71, 97, 98], qualifications or credentials [13, 99, 100] and language competence [50, 71].

Some older job seekers were keen to learn new skills that would help them obtain paid work [46], particularly technology training [71]. Older employees who wanted to develop their skills and knowledge were less likely to retire [60], and more likely to ‘unretire' and return to the workforce [65]. Additional training during midlife weakened early retirement intentions [58], and extended working lives for women more than for men [13]. De Grip et al. [101] found employees who had training opportunities and believed their employer was investing in their future employability worked 6 months longer than those without training opportunities and 5 months longer than employees with training opportunities who did not have the same strong belief of reciprocity.

##### 2.4.1.4. Older Workers' Employment History

The number of years an individual has been employed before age 50 [87, 102], and their length of job tenure with a single employer [88] is linked to individual's decision to retire. In contrast, the proportion of years a worker was unemployed strongly correlated with involuntary unemployment in later life [77]. Typically, a longer employment record was found to result in earlier eligibility for pensions and a hastened transition to retirement; however, career interruptions alone did not appear to have an association with the timing of retirement [87].

Entering the labour market at an older age, obtaining additional training during midlife and changing employers before age 50 lowered early retirement intentions [58]. Employees who worked part-time before age 50 were more likely to retire early than those who maintained full-time employment [58, 103]. Working in a high-status job or in a supervisory position prior to retirement was also associated with a greater likelihood of finding bridge employment (i.e., a paid job role a person has while receiving pension benefits), whereas involuntary career exit reduced the likelihood of being successful in finding bridge employment [104].

Older workers who became unemployed after age 50, and were successful in returning to paid employment, often had more stable job histories over their working lives compared to those who were still seeking employment [93]. In terms of helping older job seekers obtain employment, interventions that improved self-presentation, boosted self-efficacy and encouraged proactivity were more effective than interventions that excluded such personal development components [105].

##### 2.4.1.5. Older Workers' Lifestyle Preferences

Decisions to retire, or even retire early, were sometimes associated with lifestyle preferences [106], and a desire to have more leisure time [13, 34, 59, 107, 108]. Yet, the freedom that comes with retirement is not always welcomed by some older adults. Older workers who retired were more likely to re-enter the labour force if they missed the routines, responsibilities and social interactions that were involved in their previous job [65]. Extending working life was higher among those aged 66 to 71 who did not have engaging activities outside the home or were not interested in doing household chores [70].

##### 2.4.1.6. Older Workers' Personal Characteristics

Diversity among older workers also arises from individual differences in locus of control, work attitudes, adaptability, life events and career stage [109]. Other sociodemographic attributes of older workers, such as gender, immigration status and being a person of colour, can result in inequities among older workers' employment experiences. Women were more likely to work part-time as they grew older [56], settle for part-time employment despite wanting full-time employment [86], reduce workforce participation [110] and leave the labour market earlier than men [13, 90, 103, 111]. Men were more prone to retire involuntarily [13], return to work following an illness [79], work beyond typical retirement age [32, 69, 112] and choose when to leave paid employment [77].

Compared to people born in the country in which they live, immigrants were more likely to retire involuntarily [83, 113], experience long-term unemployment [84], and be less likely to retire early [31]. Similarly, older workers who are persons of colour were more vulnerable to involuntary labour force exits [114]. In a large U.S. longitudinal study, Brown and Warner [85] found that the odds of being retired for older women who were Black or Hispanic were higher than for women who were White. They concluded that racial and ethnic differences in mid- and later-life work behaviour stemmed from cumulative disadvantage across the life course. Women with less human capital, without access to employer-provided pensions, with less household wealth, who were divorced or widowed and who were in poorer health were more likely to exit the labour force involuntarily. Similarly, older, disadvantaged and low-skilled job seekers who had undertaken training activities to increase their employability were unable to overcome the barriers of societal and structural disadvantages accumulated over their life course to find suitable employment [115].

#### 2.4.2. Context 2: Family Context of Older Workers

##### 2.4.2.1. Older Workers' Obligations With Intimate Partners

The association between marital status and older workers' employability is complicated and often contradictory. Differentiations occurred in relation to whether or not an older worker has an intimate partner (spouse or common law) and, if they have a spouse or partner, whether or not their spouse/partner is employed, and the quality of their relationship. Gender and the experience of major life course transitions (such as widowhood and divorce) also influence older workers' employability.

Some studies found differences in the labour force attachment between older workers who were single and those who were coupled. Compared to those who had a partner, older workers who were unattached were more likely to delay their retirement [116], work beyond the retirement age of 65 [69] or otherwise extend their working lives [13]. The exception being those who had recently become widowed; they were more likely to have left the labour force before the usual retirement age, likely associated with responsibilities of caring for a dying partner [68, 78, 117–119].

Among older workers who were married, the evidence is inconclusive. Some studies found that early retirees are often living together with a partner [87, 94, 101, 112, 116], and may leave the labour force early because of income pooling [87]. In contrast, other studies found that having a partner decreases the likelihood of early retirement and unemployment [29] and prolongs the labour force participation of older workers to time retirement with younger partners [92]. Other studies found that marital status was not a significant predictor of exiting paid work [32, 120, 121].

The influence of a spouse or partner's labour force participation on older workers' employment is also inconclusive. Some studies found that older workers whose spouses are working are more likely to retire early [77, 95]. In contrast, other studies found the opposite: older workers whose spouses are working were more likely to be employed [122] and less likely to detach from the labour force [31], either by choice or for involuntary reasons [77]. Yet, other studies found that having a partner who is retired or otherwise not employed increased the likelihood of early labour force exit among older workers [13, 52, 74, 88, 118, 123]. Some older workers time their labour force exit to coincide with their spouse's exit [124]. Only one study found no difference in retirement intentions between dual earner couples and that of older workers with a nonworking spouse [116].

For older women in particular, marital status has varying implications for their labour force attachment. Older women are more likely than men to cite family reasons for their unemployment [50] and retirement [13]. Several studies found that older women who are married or cohabitating are more likely to retire early [34, 78, 85, 112], especially if their spouses are still working [125]. In contrast, one study found married women aged 55 and older were more likely to be in the labour force compared to single women of the same age [117].

While the relationship between the marital status of older workers and their employability is mixed, marital quality influences the labour force participation of coupled older workers. Older employed couples who have a high level of shared leisure activities, want to spend more time together, and have a positive attitude towards retiring are more likely to leave the paid labour force [60, 62, 126]. A partner's personal desire for their employed spouse to retire can also influence retirement timing [108].

Finally, the financial consequences associated with divorce forces some older workers to work longer or seek re-employment to catch up financially [58]. Overall, older men and women who have been separated or divorced are more likely than individuals who are married or have never married to seek re-employment after leaving a long-term job of 12 years or more [127]. Extending working lives is especially common among women who become divorced or widowed in midlife [102, 128] and men who divorce after age 45 [58].

##### 2.4.2.2. Older Workers' Obligations to Dependent Children

Older workers who have dependent children are more likely to remain employed in later life [93] and delay their retirement [112]. Both men and women with children expect to work longer because of the financial costs of raising children than those without children [129, 130], although caring for children may limit the number of hours available to work for pay [131]. For example, men who have their first child after age 30 intend to retire later than those who had their first child between the ages of 24 and 29 [58]. Furthermore, larger family sizes decrease the likelihood of early retirement [78]. Becoming a grandparent is associated with leaving the paid labour force before age 65 among older women of higher socioeconomic status [132].

##### 2.4.2.3. Older Workers' Caregiving Obligations

In addition to caring for dependent children, providing care to family members who have chronic health conditions, physical or mental disabilities or ageing-related needs also competes with older workers' ability to work for pay. Persons aged 50 to 64 have the highest rates of personal caregiving responsibilities among all age groups [133], with caregiving more common among women than men [102, 134].

Overall, evidence suggests that the presence of caregiving responsibilities contributes to greater absenteeism, stress and disruptions at work [1], and restricts labour force participation [135], forces early retirement [34, 62] and otherwise inhibits the extension of working lives of older workers [13]. Having high levels of care demands in particular contributes to greater work–life dissatisfaction [136, 137], working fewer hours for pay [131], early labour force exits [138] and being viewed negatively by employers [139]. Among older workers with care responsibilities, the availability of flexible work options is key to helping them integrate their paid work and care responsibilities [140] and maintain their labour force attachment [141], particularly among older women [142].

#### 2.4.3. Context 3: Workplace Context of Older Workers

##### 2.4.3.1. Organizational Characteristics of Older Workers' Employer

Findings about how company size affects the labour force participation of older workers are mixed. Several articles highlighted the differences in the ability between small and medium enterprises and large companies to support the employability of older workers [136, 143]. Smaller firms may not have the capacity to provide ongoing education and training [144] nor provide flexible work arrangements for older employees [145]. In contrast, larger organizations with dedicated human resource departments may be able to provide better workplace accommodations for older employees than smaller firms [1, 97]. During times of economic downturns or organizational restructuring, older workers were more likely than younger workers to have their jobs displaced [146, 147]. In contrast, when an organization experiences shortages in their skilled workforce, older job seekers were more likely to be recruited, hired and retained [147, 148].

##### 2.4.3.2. Workplace Policies and Programs Available to Older Workers

Many older workers would stay employed longer if they had access to more flexible work arrangements [108]. Highly desirable work arrangements among older workers includes determining their working hours [149, 150], work remotely or a compressed work week [151], being able to work part-time [71, 152] and having a variety of work tasks [42].

Access to ongoing education, on-the-job training and learning opportunities supports the employability of older workers [63, 150, 153–155] and increases their desire to remain working with their employer [156, 157]. However, employers in rural and remote regions may have fewer resources than those in urban areas to provide employment education and training supports [158]. Workplace programs that encourage healthier lifestyles (e.g., good nutrition, smoking cessation) can improve the overall health of older workers [159], and in turn, extend their working lives [160, 161].

##### 2.4.3.3. Characteristics of Older Workers' Jobs

The type of work involved in an occupation affected the employability of older workers. Jobs with high physical demands, often referred to as ‘arduous jobs' [154], were commonly reported to be unsustainable for some workers as they grew older [62, 138, 162]. More specifically, jobs that have high physical strain or high need for recovery after a day of work prevented older workers from continuing such jobs into later life [150, 163], contributed to early retirement [164, 165] and increased the likelihood of retiring because of an acquired disability [120]. Workers in arduous jobs often had lower levels of education, making the transition difficult to other jobs that are less physically demanding [162].

Jobs that have interesting and challenging aspects to them motivated some older workers to continue working [58, 69, 108, 116, 125, 159, 166, 167]. Those who work because they liked to work and those with higher levels of engagement often worked beyond the retirement age of 65 [69]. In contrast, older workers with low levels of job satisfaction [111, 157, 165, 167] or who worked in positions with low job control [49, 53, 80, 168] or low decision-making autonomy [138, 169] were less likely to extend their working lives. Furthermore, older workers who were disengaged from work prior to retirement [104] and those who increased their life satisfaction after retirement [72] were less likely to return to the paid labour force.

##### 2.4.3.4. Older Workers' Workplace Relationships

The quality of the social atmosphere at work and having good relationships with colleagues and managers greatly affects the employability of older workers. Older workers with high levels of workplace support reported greater commitment, job satisfaction and lower turnover intentions compared to those with low levels of support [170]. Older workers who trusted their supervisors or had good relationships with co-workers were more likely to continue working [13, 60, 69, 78, 90, 108, 159, 171–174] and less likely to have strong retirement intentions [173]. Older workers who received recognition and appreciation for their work were also more inclined to stay with their employer [60, 107, 151, 159, 169, 175–177], and work beyond age 65 [108]. Older workers appreciated meaningful opportunities to help others [71] and mentor younger colleagues, which contributed to their sense of belonging to an organization [70].

In contrast, toxic workplace politics, aggression and conflicts drove some older workers to leave their jobs before age 65 [57, 59, 62]. Poor relationships with co-workers [133] and a lack of respect, recognition and appreciation from managers [60, 107, 169] contributed to burnout [126] and increased older workers' desire to leave their current positions and retire early [164, 165, 178]. Lack of social support was also a barrier to returning to work after recovery from a major health event [79] and affected older adults' ability to secure a new job after becoming unemployed [93].

#### 2.4.4. Context 4: Broader Sociopolitical and Historical Context in Which Older Workers Live

##### 2.4.4.1. Ageism

Ageism, the negative attitudes, beliefs and stereotypes about a person or group based on their known or perceived age, has been recognized as a major barrier to the employability of older workers in Australia [135, 179], Canada [141, 180], Europe [150, 181], the United States [99, 155] and throughout the countries that comprise the OECD [143, 182]. Almost all OECD countries have human rights legislation that prohibits discrimination based on age [182], yet ageism remains ubiquitous. Laws intended to protect against age discrimination may not influence workplace cultures within organizations, nor buffer older workers against the effects of ageist beliefs and behaviours [143]. Countries like Australia, Finland, France, the Netherlands, Norway and the United Kingdom have undertaken information campaigns to educate employers on how to better manage and support an older workforce [182]. While there is recognition among policymakers that more needs to be done to raise awareness about ageism and address ageist beliefs in society, key contexts for such change are labour markets and workplaces [150, 181, 183].

Overall, younger and older workers tend to report experiencing age discrimination more than middle-aged workers [140, 181]. Among older workers who experience age discrimination, workers with lower levels of education and skills report experiencing less age discrimination [140]. Older workers themselves may also believe negative stereotypes as an inevitable part of the ageing process [141, 183].

Age-related stereotypes among employers contributes to myths that older workers are less productive [73, 152, 183], resistant to learning new skills [180] and overall do not provide a good return on investment [139]. Furthermore, ageist beliefs held by managers negatively impact the selection and hiring processes for older job seekers [99, 184, 185]; [138, 183] as well as their decision-making about the career pathways of older workers under their supervision and when they ‘should' retire [183]. The ‘double jeopardy' of gendered ageism in the workplace highlights challenges older female job seekers face beyond having sufficient skills to ‘more intangible manifestations of gendered ageism' [75]. Biases based on appearance, perceived lack of team fit and beliefs that younger managers may perceive older workers as a threat to their authority made it difficult to secure permanent employment (in the clerical labour market) and eroded older women's self-confidence in the job search process.

##### 2.4.4.2. Government Labour and Pension Policies

Over the last two decades, governments of high-income countries have removed mandatory retirement age legislation [143] and reformed their pension and retirement systems to promote the continued labour force attachment of older workers [150, 183]. There are two main policy goals underlying public pension system reform: to prevent future labour shortages and to ensure the sustainability of social programs [145]. Among countries in the European Union, changes to pension systems to extend working lives include financial penalties for early withdrawals and bonuses for individuals who defer accessing a public pension [145]. In the United Kingdom, the recent policy shift to harmonize the retirement ages of men and women, gradually raising the official retirement age for women from 60 to 65, has created retirement planning hardships, particularly for those with poor health [100]. Other reforms in Europe [133] and Canada [150, 183] include offering flexible or partial retirement schemes that allow older people to draw income from their pensions while remaining in the labour force. While government policies related to early retirement have been scaled back in recent years, the use of early retirement schemes and practices by employers still exist in many high-income countries [143]. Trade and labour unions, especially in Europe, established early workforce exit pathways for older workers in their membership [112, 186] that may not allow workers to return to the jobs they left after they retire [187].

##### 2.4.4.3. Macroeconomic Conditions

The global recession caused by the 2007–2008 financial crisis, often called the Great Recession, had a significant impact on economies and labour force participation rates in high-income countries. Overall, older workers faired relatively well during the Great Recession, partly because they were better educated than previous generations of older workers [145, 149, 185]. Despite this, many European employers still used early retirement schemes targeted toward older workers to reduce staffing and mitigate financial issues during the crisis [149]. In Canada, older workers with less than a four-year university degree experienced worse employment outcomes overall during the Great Recession [1], while in the United States, the labour market participation of older workers increased following the financial crisis [188].

## 3. Discussion

This scoping review builds upon a previous systematic mapping review conducted by Edge et al. [13] by examining the breadth of global literature on barriers and facilitators of older worker employability. Our review drew on a human ecological model [25] that emplaces workers aged 50 and older within assemblages constituted of individual, familial, work and societal environments and relationships. This review suggests the ability of older workers' to secure or maintain employment is not attributable to a single factor within any of these contexts, a finding echoed in previous research [62]. While a scoping review provides a useful conceptual map, it cannot be taken as a state-of-knowledge treatise on all aspects of a phenomenon. Our findings are notably focused on publications from North America, Europe and Oceania, with limited studies from Asia and Latin America, and no studies from the entire African continent identified. This limitation may derive from excluding publications not written in English, a methodological decision that may skew the focus towards high-income countries in the Global North.

The reviewed studies largely excluded the COVID-19 pandemic. We decided not to include studies explicitly focused on the labour force impact of the virus as this was an emerging area of research during our literature retrieval period before vaccines were widely available. Recent employment phenomena resulting from the pandemic, like the mass exodus of certain workers known as The Great Resignation, appeared to have led some older workers to retire early after a period of unemployment [189]. However, the long-term consequences for this behavioural shift are yet to be determined.

The variegated experiences of older workers may require policies, developed by both governments and employers, that recognise how individual workers' dynamic, lived experiences and obligations—within families, workplaces and society—shape their ability to maintain paid employment in later life. Familial and care obligations can significantly disrupt older individuals' employment, resulting in increased absenteeism, reduced paid work hours and withdrawal from the workforce. These relationships can pose particular issues for older women's financial (in)security and well-being given the gendered impacts of kin-based care work. Employers capable of supporting individuals with these care responsibilities can prevent turnover and the potential labour force withdrawal of older workers [190].

We recommend supporting approaches within social policy that target employment and ageing-related policies and where they intertwine. As all workers will likely experience paid work interruptions due to personal health issues and kin-based care responsibilities, capturing more holistic accounts of supportive workplace environments will help inform policies and practices that sustain the labour force participation of older workers. This relational approach may particularly help to identify specific support required for people from disadvantaged backgrounds to help them maintain continuous employment throughout the life course, including later life. After all, older workers have accumulated a lifetime of skills and experience that should be seen as an asset to individual companies and national economies rather than entrenching ageist imaginaries about older workers as liabilities.

Future research will need to explore local experiences of working in later life, especially in the Global South, as only about one in 10 articles included were from that region. Public pension systems in the Global South are not nearly as robust as systems in the Global North [191], which implies older workers will likely be more reliant on employment income to support their financial well-being. Furthermore, our scoping review identified little research on older people from ethnically minoritized and other marginalized backgrounds and none from outside the United States [85, 114]. Future research must explore the intersectionalities of ethnicity, gender, sexuality and other protected characteristics in association with a person's age-related experiences. Such research may require the development of new large-scale datasets sensitized to these characteristics within multiple national contexts to understand the scope of challenges for older people from these backgrounds seeking to maintain employment.

We also recommend that ageing policy align more closely with research on the future of work. With the increased disruption from digital platformization of work and the increasing prominence of gig work [192], how future generations age with increasingly precarious employment will also require additional consideration [193]. The impacts that automation and artificial intelligence will have on global workforces is not yet apparent. On one hand, workplace relations between employers/contractors and clients that are mediated through digital platforms may influence what ‘meaningful' and ‘good' work means for older workers in ways that may hasten their departure from paid employment. On the other hand, the greater value of having experience to judge whether AI is performing sensibly for a given task is becoming more valuable, which may open new opportunities for older workers. Whether positive or negative, significant disruptions to working life through events such as pandemics and major technology shifts cannot be viewed in isolation from an ageing workforce.

Our human ecology framework provided a theoretical basis for this scoping review that could be used to frame future empirical research. Social scientists seek to contextualize individual experiences and biographies within the social, political and historical context within which they are situated, an approach characterized by the ‘sociological imagination' [194]. The development of a ‘gerontological imagination', which situates ageing within its social, cultural, political and economic milieux, may help scholars interrogate the relationships between the agency of individuals as they age and the social structures that may support or inhibit their activities and aspirations as they grow older, including decisions to maintain employment.

## 4. Conclusion

This scoping review explored the dynamic ecology that influences the employability of people in later life. Older workers' abilities to maintain their labour force participation are fluid, situational and temporal, including aspects that arise across a person's life course as forms of cumulative advantage and disadvantage. Societies are fundamentally breaching the social contract of working lives where workers provide decades of labour for a secure retirement in later life. Policymakers have done little to address decreases in social protections as countries plan for future increases to retirement ages. The extension of working lives will be inequitable if provisions are not made to support older workers that have had physically demanding work histories or have health issues that limit their labour force participation. Given the realities of population ageing, workers are likely to experience employment interruptions associated with care and caregiving responsibilities. The design of future jobs will need to better accommodate these unpaid yet valuable family roles and support opportunities for career transitions and reskilling for older workers in ways that do not jeopardize their financial well-being.

## Figures and Tables

**Table 1:** Literature search terms and search strategy.

Population of interest Mature workers or job seekers aged 50+	(S1) Ageing employee^∗^ or ageing job seeker^∗^ or ageing worker^∗^ or ageing employee^∗^ or ageing job seeker^∗^ or ageing worker^∗^ or mature employee^∗^ or mature job seeker^∗^ or mature worker^∗^ or older employee^∗^ or older job seeker^∗^ or older worker^∗^

Concept Factors that enable or impede employment	(S2) Enable^∗^ or facilitat^∗^ or opportunit^∗^ (S3) Barrier^∗^ or challenge^∗^ or difficult^∗^ (S4) Risk factor^∗^ or contributing factor^∗^ or influencing factor^∗^ or push factor^∗^ or predictor^∗^

Context Paid employment, involuntary labour force exit, or job re-entry	(S5) Employability or employ^∗^ or employment reentry or job^∗^ or paid work or labour force participat^∗^ or labour force participat^∗^ or labour market attach^∗^ or labour market attach^∗^ or workforce participat^∗^ or workforce attach^∗^ (S6) Early retire^∗^ or involuntary retire^∗^ or labour force exit or labour force exit or unemploy^∗^ or layoff^∗^ or terminat^∗^ or redundan^∗^

Final search Strategy string	S1 AND (S2 OR S3 OR S4) AND (S5 AND S6)

**Table 2:** Overview of findings represented by context.

Context	Codes	Frequency count (*n* = 244)	Proportion of articles included
Individual	• Health • Income and wealth accumulation • Education and skills • Employment history • Lifestyle preferences • Personal characteristics	231	94.7%
Family	• Obligations with intimate partners • Obligations to support dependent children • Caregiving obligations and responsibilities	94	38.5%
Workplace	• Organizational characteristics • Workplace policies and programs • Job characteristics • Workplace relationships	166	68.0%
Broader sociopolitical and historical	• Ageism • Government labour and pension policies • Macro economic conditions	82	33.6%

*Note:* Frequencies and percentages will exceed 244 articles and 100%, respectively, as included articles can have findings associated with more than one context.

## Data Availability

This manuscript does not report on data. Data extraction files used for analysis can be made available upon reasonable request.

## References

[1] National Seniors Council (2013). Older Workers at Risk of Withdrawing From the Labour Force or Becoming Unemployed: Employers’ Views on How to Retain and Attract Older Workers.

[2] United Nations (2020). The Sustainable Development Goals Report 2020.

[3] United Nations (2017). Leaving No One Behind: Equality and Non-discrimination at the Heart of Sustainable Development.

[4] Butler R. N. (2008). *The Longevity Revolution: The Benefits and Challenges of Living a Long Life*.

[5] OECD (2024). OECD Better Life Index.

[6] Statistics Canada (2022). A Portrait of Canada’s Growing Population Aged 85 and Older from the 2021 Census.

[7] Buchholz K. (2021). There Are now More than Half a Million People Aged 100 or Older Around the World.

[8] Gee E. M. T., Gutman G. M. (2000). *The Overselling of Population Aging: Apocalyptic Demography, Intergenerational Challenges, and Social Policy*.

[9] Ní Léime Á., Ogg J., Rašticová M. (2020). *Extended Working Life Policies: International Gender and Health Perspectives*.

[10] Davey J. (2014). Age Discrimination in the Workplace. *Policy Quarterly*.

[11] Berkman L. F., Boersch-Supan A., Avendano M. (2015). Labor-Force Participation, Policies & Practices in an Aging America: Adaptation Essential for a Healthy & Resilient Population. *Dædalus*.

[12] Centre for Ageing Better (2023). Conditions Not Met for Further Acceleration of State Pension Age Increases.

[13] Edge C. E., Cooper A. M., Coffey M. (2017). Barriers and Facilitators to Extended Working Lives in Europe: a Gender Focus. *Public Health Reviews*.

[14] Staudinger U. M., Finkelstein R., Calvo E., Sivaramakrishnan K. (2016). A Global View on the Effects of Work on Health in Later Life. *The Gerontologist*.

[15] Guilbert L., Bernaud J.-L., Gouvernet B., Rossier J. (2016). Employability: Review and Research Prospects. *International Journal for Educational and Vocational Guidance*.

[16] Arksey H., O’Malley L. (2005). Scoping Studies: towards a Methodological Framework. *International Journal of Social Research Methodology*.

[17] Levac D., Colquhoun H., O’Brien K. K. (2010). Scoping Studies: Advancing the Methodology. *Implementation Science*.

[18] Munn Z., Pollock D., Khalil H. (2022). What Are Scoping Reviews? Providing a Formal Definition of Scoping Reviews as a Type of Evidence Synthesis. *JBI Evidence Synthesis*.

[19] Peters M. D. J., Godfrey C. M., Khalil H., McInerney P., Parker D., Soares C. B. (2015). Guidance for Conducting Systematic Scoping Reviews. *International Journal of Evidence-Based Healthcare*.

[20] Peters M. D. J., Godfrey C., McInerney P., Munn Z., Tricco A. C., Khalil H., Aromataris E., Munn Z. (2020). *Chapter 11: Scoping Reviews (2020 Version)*.

[21] Harris K., Krygsman S., Waschenko J., Laliberte Rudman D. (2018). Ageism and the Older Worker: a Scoping Review. *The Gerontologist*.

[22] Shultz K. S., Morton K. R., Weckerle J. R. (1998). The Influence of Push and Pull Factors on Voluntary and Involuntary Early Retirees’ Retirement Decision and Adjustment. *Journal of Vocational Behavior*.

[23] Tyndall J. (2008). How Low Can You Go? Towards a Hierarchy of Grey Literature.

[24] Desjardins D., Freestone C., Powell N. (2020). Pandemic Threatens Decades of Women’s Labour Force Gains.

[25] Bubolz M. M., Sontag S., Boss P. G., Doherty W. J., Larossa R., Schumm W. R., Steinmetz S. K. (1993). Human Ecology Theory. *Sourcebook of Family Theories and Methods: A Contextual Approach*.

[26] Gale N. K., Heath G., Cameron E., Rashid S., Redwood S. (2013). Using the Framework Method for the Analysis of Qualitative Data in multi-disciplinary Health Research. *BMC Medical Research Methodology*.

[27] Barnay T. (2010). In Which Ways do Unhealthy People Older than 50 Exit the Labour Market in France?. *The European Journal of Health Economics*.

[28] van Zon S. K. R., Reijneveld S. A., Galaurchi A., Mendes de Leon C. F., Almansa J., Bültmann U. (2020). Multimorbidity and the Transition out of full-time Paid Employment: a Longitudinal Analysis of the Health and Retirement Study. *The Journals of Gerontology: Series B*.

[29] Alavinia S. M., Burdorf A. (2008). Unemployment and Retirement and ill-health: a cross-sectional Analysis Across European Countries. *International Archives of Occupational and Environmental Health*.

[30] Camarano A. A., Carvalho D. F., Kanso S. (2019). Exiting the Labour Market Early: Retirement or Discrimination?. *Ciência & Saúde Coletiva*.

[31] Chen W.-H. (2019). Health and Transitions into Nonemployment and Early Retirement Among Older Workers in Canada. *Economics and Human Biology*.

[32] De Preter H., Van Looy D., Mortelmans D. (2013). Individual and Institutional Push and Pull Factors as Predictors of Retirement Timing in Europe: a Multilevel Analysis. *Journal of Aging Studies*.

[33] Bjelica B., Basta I., Bozovic I. (2018). Employment Status of Patients with Chronic Inflammatory Demyelinating Polyradiculoneuropathy. *Journal of the Peripheral Nervous System*.

[34] Boumans N. P. G., De Jong A. H. J., Vanderlinden L. (2008). Determinants of Early Retirement Intentions Among Belgian Nurses. *Journal of Advanced Nursing*.

[35] Bađun M., Smolić Š. (2018). Predictors of Early Retirement Intentions in Croatia. *Društvena Istraživanja*.

[36] Paunio T., Korhonen T., Hublin C. (2015). Poor Sleep Predicts Symptoms of Depression and Disability Retirement due to Depression. *Journal of Affective Disorders*.

[37] Axelrad H., Tur-Sinai A. (2019). Switching to self-employed when Heading for Retirement. *Journal of Applied Gerontology*.

[38] Beutel T. F., Adams J., Becker J., Letzel S., Rose D. M. (2018). Vocational Disability in Teachers – Influencing Factors Among a Highly Burdened Sample. *Journal of Vocational Rehabilitation*.

[39] Porru F., Burdorf A., Robroek S. J. W. (2019). The Impact of Depressive Symptoms on Exit from Paid Employment in Europe: a Longitudinal Study with 4 Years follow-up. *The European Journal of Public Health*.

[40] Butler S., Wardamasky S., Brennan-Ing M. (2012). Older Women Caring for Older Women: the Rewards and Challenges of the Home Care Aide Job. *Journal of Women & Aging*.

[41] Leijten F. R. M., de Wind A., van den Heuvel S. G. (2015). The Influence of Chronic Health Problems and work-related Factors on Loss of Paid Employment Among Older Workers. *Journal of Epidemiology & Community Health*.

[42] Beck V. (2013). Employers’ Use of Older Workers in the Recession. *Employee Relations*.

[43] Carmichael F., Hulme C., Porcellato L. (2013). Older Age and Ill‐Health: Links to Work and Worklessness. *International Journal of Workplace Health Management*.

[44] Arndt V., Koch-Gallenkamp L., Bertram H. (2019). Return to Work After Cancer. A multi-regional Population-based Study from Germany. *Acta Oncologica*.

[45] Virtanen M., Kivimäki M., Vahtera J. (2006). Sickness Absence as a Risk Factor for Job Termination, Unemployment, and Disability Pension Among Temporary and Permanent Employees. *Occupational and Environmental Medicine*.

[46] Nakai Y., Chang B., Snell A. F., Fluckinger C. D. (2011). Profiles of Mature Job Seekers: Connecting Needs and Desires to Work Characteristics. *Journal of Organizational Behavior*.

[47] Angeloni S., Borgonovi E. (2016). An Ageing World and the Challenges for a Model of Sustainable Social Change. *The Journal of Management Development*.

[48] Crawford J. O., Graveling R. A., Cowie H. A., Dixon K. (2010). The Health Safety and Health Promotion Needs of Older Workers. *Occupational Medicine*.

[49] van den Berg T., Schuring M., Avendano M., Mackenbach J., Burdorf A. (2010). The Impact of Ill Health on Exit from Paid Employment in Europe Among Older Workers. *Occupational and Environmental Medicine*.

[50] Crăciun I. C., Rasche S., Flick U., Hirseland A. (2019). Too Old to Work: Views on Reemployment in Older Unemployed Immigrants in Germany. *Ageing International*.

[51] Szubert Z., Sobala W. (2005). Current Determinants of Early Retirement Among Blue Collar Workers in Poland. *International Journal of Occupational Medicine & Environmental Health*.

[52] Rice N. E., Lang I. A., Henley W., Melzer D. (2011). Common Health Predictors of Early Retirement: Findings from the English Longitudinal Study of Ageing. *Age and Ageing*.

[53] Robroek S. J. W., Schuring M., Croezen S., Stattin M., Burdorf A. (2013). Poor Health, Unhealthy Behaviors, and Unfavorable Work Characteristics Influence Pathways of Exit from Paid Employment Among Older Workers in Europe: a Four Year follow-up Study. *Scandinavian Journal of Work, Environment & Health*.

[54] Morois S., Lemogne C., Leclerc A. (2016). More than Light Alcohol Consumption Predicts Early Cessation from Employment in French middle-aged Men. *Alcohol and Alcoholism*.

[55] Roy S. B. (2018). Effect of Health on Retirement of Older Americans: a Competing Risks Study. *Journal of Labor Research*.

[56] Choi C., Yu P. (2015). Why do Australians Retire Early or Late? an Analysis of a National Longitudinal Dataset. *American Journal of Medical Research*.

[57] Conn L. G., Wright F. C. (2018). Retirement Plans and Perspectives Among General Surgeons: a Qualitative Assessment. *Canadian Journal of Surgery*.

[58] Damman M., Henkens K., Kalmijn M. (2011). The Impact of Midlife Educational, Work, Health, and Family Experiences on Men’s Early Retirement. *Journals of Gerontology Series B: Psychological Sciences and Social Sciences*.

[59] de Wind A., Geuskens G. A., Reeuwijk K. G. (2013). Pathways Through Which Health Influences Early Retirement: a Qualitative Study. *BMC Public Health*.

[60] de Wind A., Geuskens G. A., Ybema J. F. (2014). Health, Job Characteristics, Skills, and Social and Financial Factors in Relation to Early Retirement-Results from a Longitudinal Study in the Netherlands. *Scandinavian Journal of Work, Environment & Health*.

[61] Kjær T., Bøje C. R., Olsen M. H. (2013). Affiliation to the Work Market After Curative Treatment of head-and-neck Cancer: a Population-based Study from the DAHANCA Database. *Acta Oncologica*.

[62] Reeuwijk K. G., de Wind A., Westerman M. J., Ybema J. F., van der Beek A. J., Geuskens G. A. (2013). ‘All Those Things Together Made me Retire’: Qualitative Study on Early Retirement Among Dutch Employees: Qualitative Study on Early Retirement Among Dutch Employees. *BMC Public Health*.

[63] Le Blanc P. M., Peeters M. C. W., Van der Heijden B. I. J. M., van Zyl L. E. (2019). To Leave or Not to Leave? A multi-sample Study on Individual, job-related, and Organizational Antecedents of Employability and Retirement Intentions. *Frontiers in Psychology*.

[64] Bell D. N. F., Rutherford A. C. (2013). Older Workers and Working Time. *The Journal of the Economics of Ageing*.

[65] Schlosser F., Zinni D., Armstrong‐Stassen M. (2012). Intention to Unretire: HR and the Boomerang Effect. *Career Development International*.

[66] Congdon-Hohman J. (2018). Retirement Reversals and Health Insurance. *Public Finance Review*.

[67] Andrews J., Manthorpe J., Watson R. (2005). Employment Transitions for Older Nurses: a Qualitative Study. *Journal of Advanced Nursing*.

[68] Schreiber P. (2018). Widowhood and Retirement Timing: Evidence from the Health and Retirement Study. *The B.E. Journal of Economic Analysis & Policy*.

[69] de Wind A., van der Pas S., Blatter B. M., van der Beek A. J. (2016). A Life Course Perspective on Working Beyond retirement-results from a Longitudinal Study in the Netherlands. *BMC Public Health*.

[70] Hovbrandt P., Håkansson C., Albin M., Carlsson G., Nilsson K. (2019). Prerequisites and Driving Forces Behind an Extended Working Life Among Older Workers. *Scandinavian Journal of Occupational Therapy*.

[71] Lee C. C., Czaja S. J., Sharit J. (2008). Training Older Workers for Technology-based Employment. *Educational Gerontology*.

[72] Armstrong‐Stassen M., Schlosser F., Zinni D. (2012). Seeking Resources: Predicting Retirees’ Return to Their Workplace. *Journal of Managerial Psychology*.

[73] Oesch D. (2020). Discrimination in the Hiring of Older Jobseekers: Combining a Survey Experiment with a Natural Experiment in Switzerland. *Research in Social Stratification and Mobility*.

[74] Friis K., Ekholm O., Hundrup Y. A., Obel E. B., Grønbæk M. (2007). Influence of Health, Lifestyle, Working Conditions, and Sociodemography on Early Retirement Among Nurses: the Danish Nurse Cohort Study. *Scandinavian Journal of Public Health*.

[75] Handy J., Davy D. (2007). Gendered Ageism: Older Women’s Experiences of Employment Agency Practices. *Asia Pacific Journal of Human Resources*.

[76] Biehl A., Gurley-Calvez T., Hill B. (2014). Self-Employment of Older Americans: Do Recessions Matter?. *Small Business Economics*.

[77] Gong C. H., He X. (2019). Factors Predicting Voluntary and Involuntary Workforce Transitions at Mature Ages: Evidence from HILDA in Australia. *International Journal of Environmental Research and Public Health*.

[78] Piłat A., Wilga M., Leonardi M., Vlachou A., Tobiasz-Adamczyk B. (2019). Challenges for the Labor Market: 2 Complementary Approaches to Premature Cessation of Occupational Activity. *International Journal of Occupational Medicine & Environmental Health*.

[79] Mehnert A. (2011). Employment and work-related Issues in Cancer Survivors. *Critical Reviews in Oncology*.

[80] Mäcken J. (2019). Work Stress Among Older Employees in Germany: Effects on Health and Retirement Age. *PLoS One*.

[81] de Breij S., Huisman M., Deeg D. J. H. (2019). Educational Differences in macro-level Determinants of Early Exit from Paid Work: a Multilevel Analysis of 14 European Countries. *European Journal of Ageing*.

[82] Siegrist J., Wahrendorf M., Von dem Knesebeck O., Jurges H., Borsch-Supan A. (2007). Quality of Work, well-being, and Intended Early Retirement of Older Employees—Baseline Results from the SHARE Study. *The European Journal of Public Health*.

[83] Denton M., Plenderleith J., Chowhan J. (2013). Health and Disability as Determinants for Involuntary Retirement of People with Disabilities. *Canadian Journal on Aging/La Revue Canadienne du Vieillissement*.

[84] Fransen K., Boussauw K., Deruyter G., De Maeyer P. (2019). The Relationship Between Transport Disadvantage and Employability: Predicting long-term Unemployment Based on Job Seekers’ Access to Suitable Job Openings in Flanders, Belgium. *Transportation Research Part A: Policy and Practice*.

[85] Brown T. H., Warner D. F. (2008). Divergent Pathways? Racial/Ethnic Differences in Older Women’s Labor Force Withdrawal. *Journals of Gerontology Series B: Psychological Sciences and Social Sciences*.

[86] Lu L. (2010). Employment Among Older Workers and Inequality of Gender and Education: Evidence from a Taiwanese National Survey. *The International Journal of Aging and Human Development*.

[87] Hank K., Korbmacher J. M. (2013). Parenthood and Retirement: Gender, Cohort, and Welfare Regime Differences. *European Societies*.

[88] Radl J. (2013). Labour Market Exit and Social Stratification in Western Europe: the Effects of Social Class and Gender on the Timing of Retirement. *European Sociological Review*.

[89] Crudden A., McDonnall M. C., Sui Z. (2018). Losing Employment: At-Risk Employed Vocational Rehabilitation Applicants with Vision Loss. *Journal of Visual Impairment & Blindness*.

[90] Fleischmann M., Carr E., Stansfeld S. A., Xue B., Head J. (2018). Can Favourable Psychosocial Working Conditions in Midlife Moderate the Risk of Work Exit for Chronically Ill Workers? A 20-year follow-up of the Whitehall II Study. *Occupational and Environmental Medicine*.

[91] Riekhoff A.-J. (2018). Institutional and socio-economic Drivers of work-to-retirement Trajectories in the Netherlands. *Ageing and Society*.

[92] Hofäcker D., Naumann E. (2015). The Emerging Trend of Work Beyond Retirement Age in Germany. Increasing Social Inequality?. *Zeitschrift für Gerontologie und Geriatrie*.

[93] Gayen K., Raeside R., McQuaid R. (2019). Social Networks, Accessed and Mobilised Social Capital and the Employment Status of Older Workers: a Case Study. *International Journal of Sociology & Social Policy*.

[94] Damkjæ L. H., Deltour I., Suppli N. P. (2011). Breast Cancer and Early Retirement: Associations with Disease Characteristics, Treatment, Comorbidity, Social Position and Participation in a six-day Rehabilitation Course in a Register-based Study in Denmark. *Acta Oncologica*.

[95] D’Amours M. (2009). Non-Standard Employment After Age 50: How Precarious is it?. *Relations Industrielles*.

[96] Fournier G., Zimmermann H., Masdonati J., Gauthier C. (2018). Job Loss in a Group of Older Canadian Workers: Challenges in the Sustainable Labour Market Reintegration Process. *Sustainability*.

[97] Grant M., Rees S., Underwood M., Froud R. (2019). Obstacles to Returning to Work with Chronic Pain: In-Depth Interviews with People Who Are off Work due to Chronic Pain and Employers. *BMC Musculoskeletal Disorders*.

[98] Goos M., Rademakers E., Röttger R. (2020). Routine-Biased Technical Change: Individual-Level Evidence from a Plant Closure. *Research Policy*.

[99] Lassus L. A. P., Lopez S., Roscigno V. J. (2015). Aging Workers and the Experience of Job Loss. *Research in Social Stratification and Mobility*.

[100] Neary J., Katikireddi S. V., Brown J., Macdonald E. B., Thomson H. (2019). Role of Age and Health in Perceptions of Returning to Work: a Qualitative Study. *BMC Public Health*.

[101] de Grip A., Fouarge D., Montizaan R., Schreurs B. (2020). Train to Retain: Training Opportunities, Positive Reciprocity, and Expected Retirement Age. *Journal of Vocational Behavior*.

[102] Wildman J. M. (2020). Life-Course Influences on Extended Working: Experiences of Women in a UK baby-boom Birth Cohort. *Work, Employment & Society*.

[103] Fechter C. (2020). The Role of Health in Flexible Working Arrangements in Germany: Avenues to a Longer Working Life?. *Zeitschrift für Gerontologie und Geriatrie*.

[104] Dingemans E., Henkens K., Solinge H. v. (2016). Access to Bridge Employment: Who Finds and Who Does Not Find Work After Retirement?. *The Gerontologist*.

[105] Liu S., Huang J. L., Wang M. (2014). Effectiveness of Job Search Interventions: a meta-analytic Review. *Psychological Bulletin*.

[106] Vo K., Forder P. M., Tavener M. (2015). Retirement, Age, Gender and Mental Health: Findings from the 45 and up Study. *Aging & Mental Health*.

[107] Blakeley J. A., Ribeiro V. E. S. (2008). Early Retirement Among Registered Nurses: Contributing Factors. *Journal of Nursing Management*.

[108] Meng A., Sundstrup E., Andersen L. L. (2020). Factors Contributing to Retirement Decisions in Denmark: Comparing Employees Who Expect to Retire Before, At, and After the State Pension Age. *International Journal of Environmental Research and Public Health*.

[109] Claes R., Heymans M. (2008). HR Professionals’ Views on Work Motivation and Retention of Older Workers: a Focus Group Study. *Career Development International*.

[110] Hogan A., O’Loughlin K., Davis A., Kendig H. (2009). Hearing Loss and Paid Employment: Australian Population Survey Findings. *International Journal of Audiology*.

[111] McPhedran S. (2012). The Labor of a Lifetime?: Health and Occupation Type as Predictors of Workforce Exit Among Older Australians. *Journal of Aging and Health*.

[112] Hofäcker D. (2015). In Line or at Odds with Active Ageing Policies? Exploring Patterns of Retirement Preferences in Europe. *Ageing and Society*.

[113] Ebbinghaus B., Radl J. (2015). Pushed out Prematurely? Comparing Objectively Forced Exits and Subjective Assessments of Involuntary Retirement Across Europe. *Research in Social Stratification and Mobility*.

[114] Choi E., Tang F., Copeland V. C. (2017). Racial/Ethnic Inequality Among Older Workers: Focusing on Whites, Blacks, and Latinos Within the Cumulative advantage/disadvantage Framework. *Journal of Social Service Research*.

[115] Meyers R. (2017). Disadvantaged Older Jobseekers and the Concept of Bounded Agency. *International Journal of Lifelong Education*.

[116] Van Solinge H., Henkens K. (2014). Work-Related Factors as Predictors in the Retirement decision-making Process of Older Workers in the Netherlands. *Ageing and Society*.

[117] Statistics Canada (2011). Retirement, Health and Employment Among Those 55 plus.

[118] Wiktorowicz J. (2017). Competencies as a Factor of Economic Deactivation: Application of Exploratory Factor Analysis. *International Journal of Social Economics*.

[119] Wilińska M., Grzenda W., Perek-Białas J. (2019). Grandmothers and Non-grandmothers in the Polish Labor Market: the Role of Family Issues. *Journal of Family Issues*.

[120] Harkonmäki K., Martikainen P., Lahelma E. (2009). Intentions to Retire, Life Dissatisfaction and the Subsequent Risk of Disability Retirement. *Scandinavian Journal of Public Health*.

[121] Lee W., Yoon J.-H., Koo J.-W., Chang S.-J., Roh J., Won J.-U. (2018). Predictors and Estimation of Risk for Early Exit from Working Life by Poor Health Among Middle and Older Aged Workers in Korea. *Scientific Reports*.

[122] Jackson N., Cochrane B., McMillan R. (2013). Workforce Participation of Older Workers as an Element of New Zealand’s Retirement Income Framework: a Review of Existing Knowledge and Data.

[123] Statistics Canada (2008). Bridge Employment.

[124] Syse A., Solem P. E., Ugreninov E., Mykletun R., Furunes T. (2014). Do Spouses Coordinate Their Work Exits? A Combined Survey and Register Analysis from Norway. *Research on Aging*.

[125] von Bonsdorff M. E., Huuhtanen P., Tuomi K., Seitsamo J. (2010). Predictors of Employees’ Early Retirement Intentions: an 11-year Longitudinal Study. *Occupational Medicine*.

[126] Henkens K., Leenders M. (2010). Burnout and Older Workers’ Intentions to Retire. *International Journal of Manpower*.

[127] Statistics Canada (2014). Employment Transitions Among Older Workers Leaving long-term Jobs: Evidence from Administrative Data.

[128] Vickerstaff S., Cox J. (2005). Retirement and Risk: the Individualisation of Retirement Experiences?. *The Sociological Review*.

[129] Szinovacz M. E., Davey A. (2005). Predictors of Perceptions of Involuntary Retirement. *The Gerontologist*.

[130] Steiber N., Kohli M. (2017). You Can’T Always Get what You Want: Actual and Preferred Ages of Retirement in Europe. *Ageing and Society*.

[131] AARP Research (2020). Underemployment in Midlife and Older Workers.

[132] Zanasi F., Sieben I., Uunk W. (2020). Work History, Economic Resources, and Women’s Labour Market Withdrawal After the Birth of the First Grandchild. *European Journal of Ageing*.

[133] Eurofound (2017). Towards age-friendly Work in Europe: a life-course Perspective on Work and Ageing from EU Agencies.

[134] Earl C., Taylor P., Williams R., Brooke E., Bimrose J., McMahon M., Watson M. (2014). Falling Between the Cracks: Older Women and Employer Policymaking. *Women’s Career Development Throughout the Lifespan: An International Exploration*.

[135] Australian Human Rights Commission (2012). Working past our 60s: Reforming Laws and Policies.

[136] European Foundation for the Improvement of Living and Working Conditions (2008). Working Conditions of an Ageing Workforce.

[137] Statistics Canada (2009). Work–Life Balance of Older Workers.

[138] Innovation Resource Center (2020). Work for Tomorrow: Innovating for an Ageing Workforce.

[139] Professionals Australia (2015). Wasted Potential: Recommendations to Support mature-aged Workers.

[140] European Foundation for the Improvement of Living and Working Conditions (2006). A Guide to Good Practice in Age Management.

[141] Federal/Provincial/Territorial Ministers Responsible for Seniors (2021). Older Workers: Exploring and Addressing the Stereotypes.

[142] Burr V., Colley H. (2019). ‘I Just Felt as Though I Had to Drop Something’: the Implications of Care for Female Working Elder Carers’ Working Lives: the Implications of Care for Female Working Elder Carers’ Working Lives. *Ageing and Society*.

[143] OECD (2019). Working Better with Age.

[144] Statistics Canada (2012). Job-Related Training of Older Workers.

[145] Eurofound (2012). Flexicurity: Actions at Company Level.

[146] ARC Centre of Excellence in Population Ageing Research (2012). Mature-Age Labour Force Participation: Trends, Barriers, Incentives, and Future Potential.

[147] Tisch A. (2014). Firms’ Contribution to the Internal and External Employability of Older Employees: Evidence from Germany. *European Journal of Ageing*.

[148] Karpinska K., Henkens K., Schippers J. (2011). The Recruitment of Early Retirees: a Vignette Study of the Factors that Affect Managers’ Decisions. *Ageing and Society*.

[149] Eurofound (2012). Employment Trends and Policies for Older Workers in the Recession.

[150] Eurofound (2013). Role of Governments and Social Partners in Keeping Older Workers in the Labour Market.

[151] Public Policy Forum (2011). Canada’s Aging Workforce: a National Conference on Maximizing Employment Opportunities for Mature Workers.

[152] (2013). Standing Committee on Human Resources, Skills and Social Development and the Status of Persons with Disabilities.

[153] City & Guilds Centre for Skills Development (2011). Older Learners in the Workplace.

[154] Eurofound (2017). Working Conditions of Workers of Different Ages: European Working Conditions Survey 2015.

[155] AARP Public Policy Institute (2018). An Aging Labor Force and the Challenges of 65+ Jobseekers.

[156] Herrbach O., Mignonac K., Vandenberghe C., Negrini A. (2009). Perceived HRM Practices, Organizational Commitment, and Voluntary Early Retirement Among late-career Managers. *Human Resource Management*.

[157] Thorsen S. V., Jensen P. H., Bjørner J. B. (2016). Psychosocial Work Environment and Retirement Age: a Prospective Study of 1876 Senior Employees. *International Archives of Occupational and Environmental Health*.

[158] OECD (2015). Local Economic Strategies for Ageing Labour Markets: the Canadian Targeted Initiative for Older Workers in Fort St. James, British Columbia.

[159] Proper K. I., Deeg D. J. H., van der Beek A. J. (2009). Challenges at Work and Financial Rewards to Stimulate Longer Workforce Participation. *Human Resources for Health*.

[160] European Foundation for the Improvement of Living and Working Conditions (2006). Employment Initiatives for an Ageing Workforce in the EU15.

[161] Avendano M., Cylus J. (2019). Working at Older Ages: Why It’s Important, How it Affects Health, and the Policy Options to Support Health Capacity for Work.

[162] Sonnega A., Helppie-McFall B., Hudomiet P., Willis R. J., Fisher G. G. (2017). A Comparison of Subjective and Objective Job Demands and Fit with Personal Resources as Predictors of Retirement Timing in a National U.S. Sample. *Work, Aging and Retirement*.

[163] Eurofound (2012). Sustainable Work and the Ageing Workforce.

[164] Statistics Canada (2010). Health Factors and Early Retirement Among Older Workers.

[165] Sewdas R., Thorsen S. V., Boot C. R. L., Bjørner J. B., van der Beek A. J. (2020). Determinants of Voluntary Early Retirement for Older Workers with and Without Chronic Diseases: a Danish Prospective Study. *Scandinavian Journal of Public Health*.

[166] Soidre T. (2005). Retirement-Age Preferences of Women and Men Aged 55–64 Years in Sweden. *Ageing and Society*.

[167] Olesen S. C., Butterworth P., Rodgers B. (2012). Is Poor Mental Health a Risk Factor for Retirement? Findings from a Longitudinal Population Survey. *Social Psychiatry and Psychiatric Epidemiology*.

[168] Heponiemi T., Kouvonen A., Vanska J. (2008). Health, Psychosocial Factors and Retirement Intentions Among Finnish Physicians. *Occupational Medicine*.

[169] Carr E., Hagger-Johnson G., Head J. (2016). Working Conditions as Predictors of Retirement Intentions and Exit from Paid Employment: a 10-year follow-up of the English Longitudinal Study of Ageing. *European Journal of Ageing*.

[170] Brough P., Johnson G., Drummond S., Pennisi S., Timms C. (2011). Comparisons of Cognitive Ability and Job Attitudes of Older and Younger Workers. *Equality, Diversity and Inclusion: An International Journal*.

[171] Pillay H., Kelly K., Tones M. (2008). Exploring Work and Development Options to Reduce Early Labour Force Exit of Mature Aged Australians. *International Journal of Training Research*.

[172] Winkelmann‐Gleed A. (2011). Retirement or Committed to Work? Conceptualising Prolonged Labour Market Participation Through Organisational Commitment. *Employee Relations*.

[173] Muurinen C., Laine M., Pentti J. (2014). Vertical and Horizontal Trust at Work as Predictors of Retirement Intentions: the Finnish Public Sector Study. *PLoS One*.

[174] Stansfeld S. A., Carr E., Smuk M. (2018). Mid-Life Psychosocial Work Environment as a Predictor of Work Exit by Age 50. *PLoS One*.

[175] Maurits E. E. M., de Veer A. J. E., van der Hoek L. S., Francke A. L. (2015). Factors Associated with the self-perceived Ability of Nursing Staff to Remain Working Until Retirement: a Questionnaire Survey. *BMC Health Services Research*.

[176] Schröder H., Higo M., Flynn M. (2016). Workplace Accommodation for Older Teachers in Japan and Germany: the Role of the Institutional Context in Supporting Late Career Options for Teachers with Ill Health. *Management Revu*.

[177] Nova Scotia Centre on Aging (2018). Older Worker Employment and Labour Force Participation-Phase 2.

[178] Pit S. W., Hansen V. (2014). Factors Influencing Early Retirement Intentions in Australian Rural General Practitioners. *Occupational Medicine*.

[179] National Seniors Productive Ageing Centre (2013). Age Discrimination in the Labour Market: Experiences and Perceptions of Mature Age Australians.

[180] Expert Panel on Older Workers (2008). Supporting and Engaging Older Workers in the New Economy.

[181] Eurofound (2020). Role of Social Partners in Tackling Discrimination at Work.

[182] OECD (2011). Helping Older Workers Find and Retain Jobs. *Pensions at a Glance 2011: Retirement-Income Systems in OECD and G20 Countries*.

[183] Federal/Provincial/Territorial Ministers Responsible for Seniors (2018). Promoting the Labour Force Participation of Older Canadians – Promising Initiatives.

[184] OECD (2006). Live Longer, Work Longer, Ageing and Employment Policies.

[185] OECD (2013). OECD Employment Outlook 2013.

[186] van Dalen H. P., Henkens K., Wang M. (2015). Recharging or Retiring Older Workers? Uncovering the Age-based Strategies of European Employers. *The Gerontologist*.

[187] Principi A., Bauknecht J., Di Rosa M., Socci M. (2020). Employees’ Longer Working Lives in Europe: Drivers and Barriers in Companies. *International Journal of Environmental Research and Public Health*.

[188] United States Government Accountability Office (2012). Unemployed Older Workers: Many Experience Challenges Regaining Employment and Face Reduced Retirement Security.

[189] Schuster B., Radpour S., Conway E., Ghilarducci T. (2022). No “Great Resignation” for Older Workers—Mass Job Loss Drove the Retirement Surge. *Schwartz Center for Economic Policy Analysis Working Paper*.

[190] Williams A., Ding R., Lyeo J. S. (2025). Retrospective Review of a carer-employee Workplace Intervention. *Evaluation and Program Planning*.

[191] Ebbinghaus B., Karner C., Hofäcker D. (2023). Pension Governance in a Globalising World. *Research Handbook on the Sociology of Globalization*.

[192] Kaine S., Josserand E. (2019). The Organisation and Experience of Work in the Gig Economy. *Journal of Industrial Relations*.

[193] Ogg J., Rašticová M., Ní Léime Á., Ogg J., Rašticová M. (2020). Introduction: Key Issues and Policies for Extending Working Life. *Extended Working Life Policies: International Gender and Health Perspectives*.

[194] Mills C. W. (1959). *The Sociological Imagination*.

